# The Etiopathogenesis of Kawasaki Disease: Evolving Understanding of Diverse Triggers

**DOI:** 10.1002/iid3.70267

**Published:** 2025-09-28

**Authors:** Toshiro Hara, Yasunari Sakai

**Affiliations:** ^1^ Reiwa Health Sciences University Fukuoka Japan; ^2^ Kawasaki Disease Center, Fukuoka Children's Hospital Fukuoka Japan; ^3^ Kyushu University Fukuoka Japan; ^4^ Department of Pediatrics, Graduate School of Medical Sciences Kyushu University Fukuoka Japan

**Keywords:** damage‐associated molecular patterns, innate immunity, Kawasaki disease, oxidative stress, pathogen‐associated molecular patterns, pyroptosis, vasculitis

## Abstract

**Background:**

Kawasaki disease (KD) is a leading cause of acquired heart disease in children. Evidence suggests that microbial and nonmicrobial triggers for KD differ across geographical regions and environmental conditions. Although the precise triggers remain unidentified, KD is likely caused by microbial or environmental agents acting on genetically predisposed children.

**Recent Findings:**

Insights into KD pathogenesis have also been derived from three well‐established murine models, which highlight diverse vasculitis‐inducing pathways. The diversity of microbial triggers supports the hypothesis that KD arises from immune‐mediated responses rather than direct infection. Pathogen‐associated molecular patterns (PAMPs), microbe‐associated molecular patterns (MAMPs) and inflammatory cell death‐linked damage‐associated molecular patterns (DAMPs) play critical roles in KD pathogenesis. Furthermore, genetic polymorphisms associated with KD, such as *ITPKC*, *CASP3*, and *FCGR2A*, contribute to immune activation by promoting inflammasome activation, pyroptosis and antibody‐dependent enhancement (ADE), thereby intensifying inflammation. Oxidative and nitrative stress further amplify inflammatory responses, with their interplay potentially driving KD onset. The relatively low recurrence rate of KD, despite its diverse triggers, may partly be explained by the presence of anti‐DAMP antibodies. Historically, reduced exposure to infections and improved sanitation may have led to lower levels of anti‐DAMP antibodies, potentially contributing to the increased incidence of KD observed over time.

**Conclusion:**

Continued research into microbial and immune mechanisms is crucial to advance our understanding of KD pathogenesis.

## 1 Introduction

Kawasaki disease (KD) is one of the most prevalent vasculitic syndromes in childhood. Without treatment, 25%–30% of patients develop coronary artery abnormalities. With the introduction of high‐dose intravenous immunoglobulin (IVIG) and other therapies, the incidence of coronary artery lesions (CALs) has decreased. Despite these advances, KD remains a leading cause of acquired heart disease in children in developed countries [1, 2].

Following the end of the Second World War in 1945, Japan faced extremely poor sanitary conditions, resulting in widespread infectious diseases. By 1949, however, Japan had become the world's third‐largest producer of penicillin, which led to a rapid decline in infectious diseases during the 1950s. Although a few cases resembling KD were documented during this period, Dr. Kawasaki first encountered a patient with KD in 1961, and no documented cases of KD existed in Japan before 1950 [3].

Although over 50 years have passed since the initial description of KD [3], its precise etiology remains unclear. Epidemiological evidence suggests that KD arises in genetically predisposed children upon exposure to environmental or infectious triggers [1, 2, 4]. Children with KD often live in sanitized environments with reduced immunological stimulation, resulting in a less challenged immune system [4, 5]. This review discusses the etiopathogenesis of KD, with the aim of advancing future approaches to the diagnosis, treatment, and prevention of the disease.

## 2 Diversity of KD‐Like Vasculitis‐Inducing Molecules in Three Representative KD Animal Models

Three well‐established murine models of KD exist (Table 1), each demonstrating potent induction of coronary arteritis through the administration of either the *Candida albicans* water‐soluble fraction (CAWS), *Lactobacillus casei* cell wall extract (LCWE), or a nucleotide‐binding oligomerization domain‐containing protein 1 (Nod1) ligand, FK565 [6, 7, 8].

**Table 1:** Key characteristics of representative mouse models of Kawasaki disease.

	CAWS model	LCWE model	Nod1 ligand model
Triggers PAMPs [Reference]	CAWS [6]	LCWE [7]	Nod1 ligand (FK565) [8]
DAMPs	Oxidized molecules	Oxidized molecules, IL‐1α	Oxidized molecules
Others	—	Superantigen	—
PRRs and signaling pathways	Dectin‐2 Syk kinase activation CARD9‐Bcl10‐Malt1 (NF‐κB) and MAPK pathways	Tlr2 MyD88‐dependent IRAK activation NF‐κB and MAPK pathways	Nod1 RIPK2/TAK1:TAB activation NF‐κB and MAPK pathways
Innate immunity	Vascular cells Cardiac macrophages	Vascular stromal cells Macrophage, DC	Endothelial cells Cardiac macrophages
Acquired immunity	T cell/B cell‐independent	T cell‐dependent (CD8^+^ T cells)	T cell/B cell‐independent
Induction of atherosclerosis [Reference]	+ i.p. injection, *Apoe*‐KO mice [9]	+ i.p. injection, *Apoe*‐ or *Ldlr*‐KO mice [10]	+ p.o. administration, *Apoe*‐KO mice [11]

*Note:* Modified from Hara et al. [12].

Abbreviations: Apoe, apolipoprotein E; Bcl10, B‐cell lymphoma/leukemia 10; CARD9, caspase recruitment domain family member 9; CAWS, *Candida albicans* water‐soluble fraction; DAMPs, damage‐associated molecular patterns; DC, dendritic cells; i.p., intraperitoneal; IRAK, interleukin‐1 receptor‐associated kinase; KO, knockout; LCWE, *Lactobacillus casei* cell wall extract; Ldlr, low‐density lipoprotein receptor; Malt1, mucosa‐associated lymphoid tissue lymphoma translocation protein 1; MAPK, mitogen‐activated protein kinase; MyD88, myeloid differentiation factor 88; NF‐κB, Nuclear Factor kappa‐light‐chain‐enhancer of activated B cells; NLRP3, nucleotide‐binding oligomerization domain, leucine‐rich repeat and pyrin domain containing 3; Nod, nucleotide‐binding oligomerization domain‐containing protein; PAMPs, pathogen‐associated molecular patterns; p.o., per os; PRR, pattern recognition receptor; RIPK2, receptor‐interacting serine/threonine kinase 2; Scid, severe combined immunodeficiency; Syk, spleen tyrosine kinase; TAB, TAK1‐binding protein; TAK1, transforming growth factor‐β‐activated kinase 1; Tlr, toll‐like receptor.

CAWS serves as a ligand for Dectin‐2, a C‐type lectin receptor of innate immunity. CAWS induces coronary vasculitis characterized by neutrophil and monocyte infiltration, endothelial activation, and local production of pro‐inflammatory cytokines and chemokines [13]. Vasculitis induced by CAWS occurs even in T‐ and B‐cell‐deficient mice, indicating that T and B cells are not essential for vasculitis in this model [13]. The nuclear factor‐kappa B (NF‐κB) pathway and mitochondrial reactive oxygen species (ROS)‐activated Nod‐, leucine‐rich repeat‐ and pyrin domain‐containing protein (Nlrp) 3 inflammasome drive the production of interleukin (IL)‐1β in dendritic cells [14]. Additionally, the water‐soluble fraction from *Candida metapsilosis* can induce coronary arteritis and shock [15].

In the LCWE model, vasculitis induction depends on superantigens and pathogen‐associated molecular patterns (PAMPs)/microbe‐associated molecular patterns (MAMPs), which trigger the disease phenotype [7]. Key players in the pathogenesis of coronary arteritis include CD11c^+^ dendritic cells, macrophages, vascular stromal cells, T cells, and a range of cytokines [16]. Inflammasome‐mediated IL‐1β production and the release of IL‐1α, an endogenous damage‐associated molecular pattern (DAMP), are important mechanisms in this model [16]. The endothelial Nlrp3 inflammasome contributes to vascular damage in conjunction with ROS, such as nitric oxide (NO) and peroxynitrite [17]. In this model, coronary vasculitis does not develop in *Rag1*‐, *Myd88*‐, or *Tlr (toll‐like receptor)* 2‐knockout mice, or in Tlr4‐mutant C3H/HeJ mice [7, 18], and the immune cells in CALs in this model are predominantly T cells. This differs from human KD autopsy samples, where monocytes/macrophages and neutrophils predominate in early disease stages [19].

FK565 is a synthetic acyltripeptide with a molecular weight of 502.6 and works as a Nod1 ligand. It is a derivative of FK156 isolated from *Streptomyces olivaceogriseus* and acts as a PAMP in murine KD models [8]. Upon binding to Nod1, FK565 activates pro‐inflammatory signals in coronary artery vascular cells, stimulating chemokine and cytokine production [20]. These chemokines recruit monocytes, which differentiate into cardiac CD11c^+^ macrophages that drive coronary arteritis [21]. The involvement of these macrophages in the pathogenic process has been confirmed using CD11c^+^ macrophage‐deficient mice. Extended investigations have demonstrated the induction of coronary arteritis in SCID and *Rag1*‐KO mice, indicating that the acquired immune system is dispensable for the hyper‐immunoreactive conditions observed in KD vasculitis models [8, 21].

These three models, each utilizing distinct ligands and pattern recognition receptors (PRRs) such as Dectin‐2, Tlr2, and Nod1, may underscore the diversity of vasculitis‐inducing pathways in KD. KD‐like vasculitis can be induced by innate immune PAMPs/MAMPs in a T‐ and B‐cell‐independent manner, as observed in the CAWS and Nod1 ligand models. The Nlrp3 inflammasome, a central molecule in innate immunity, is crucial in both the LCWE and CAWS models. Additionally, oxidative molecules acting as DAMPs contribute to vasculitis in all models, reflecting mechanisms similar to those observed in human KD. The vasculitis‐inducing potential of CAWS varies with the structure of mannosyl linkages in *Candida* mannan, which can be influenced by culture conditions [15]. Similarly, the pathogenic activity of Nod1 ligands depends on the chemical structure of FK565 derivatives [22]. Intriguingly, all three triggers accelerate atherosclerosis in *apolipoprotein E*‐knockout mice [9, 10, 11], suggesting shared pathogenic and pathophysiological mechanisms between KD vasculitis and atherosclerosis (Table 1). In humans, various infectious microbes might have been implicated in the pathogenesis of both KD and atherosclerosis as well [1, 23].

## 3 Involvement of Microbial and Nonmicrobial Triggers in KD

Infections with microbial agents are strongly implicated as triggers for the onset of KD, based on the distinct characteristics of affected patients: age distribution (with over 80% of cases occurring in children aged 6 months to 4 years) and epidemiological patterns, including epidemics, community outbreaks, and seasonality [1]. However, it is noteworthy that there is an absence of outbreaks within households, schools, or daycare centers, and minimal evidence supporting person‐to‐person transmission. These findings suggest that microbial involvement in KD may be indirect or involve complex mechanisms. This complexity is reminiscent of the relationship between severe acute respiratory syndrome coronavirus 2 (SARS‐CoV‐2) and KD or KD‐like illness, where only a small fraction of infected individuals (approximately 1 in 1000) develops KD, despite widespread exposure [24].

Evidence supporting the diversity of KD triggers includes the following: (i) variations in KD symptoms and signs across clusters [25], (ii) IVIG resistance linked to environmental factors such as season and temperature as well as co‐occurring infections [26, 27], (iii) diversity in liquid chromatography/mass spectrometry (LC‐MS) and lipidomic profiles [28], (iv) the detection of various microbes, including bacteria and viruses, in KD patient serum samples using high‐throughput sequencing [29], and (v) transcriptomic and proteomic analyses of KD patient samples showing wide data variability [30]. We therefore summarize recent findings in the literature that provide evidence for the diverse etiopathogenesis of KD from the following three perspectives.

### 3.1 Diversity of Microbial and Nonmicrobial Triggers in KD

Various microbial agents have been associated with KD [31, 32], although their causal roles remain unconfirmed (Table 2). Among these microbes, SARS‐CoV‐2 and *Yersinia* are known to consistently cause the key KD symptoms that meet the diagnostic criteria.

**Table 2:** Exogenous triggers in Kawasaki disease.

A. Microbial triggers^a^	
a. Viruses	
DNA	Epstein–Barr virus, human adenovirus, human parvovirus B19, Torque teno virus, herpes simplex virus, human herpes virus‐6, varicella zoster virus, human bocavirus, parvovirus, and cytomegalovirus
RNA	Coxsackie virus, dengue virus, enterovirus, human coronavirus NL63, parainfluenza type 3 virus, measles virus, SARS‐CoV‐2 (severe acute respiratory syndrome coronavirus‐2), feline virus, influenza A virus H1N1, and human immunodeficiency virus
b. Bacteria	
	*Staphylococcus aureus*, *Streptococcus pyogenes*, *Streptococcus sanguinis*, Group A *Streptococcus*, *Yersinia pseudotuberculosis*, *Yersinia enterocolitica*, *Propionibacterium acnes*, *Bacillus cereus*
Mycoplasma	*Mycoplasma pneumoniae*
Chlamydia	*Chlamydia pneumoniae*
Rickettsia	*Rickettsia*‐like organisms
c. Fungi	*Candida albicans*
d. Microbial components	Lipopolysaccharides (LPS) from Gram‐negative bacteria β‐glucan from *Candida*
**B. Nonmicrobial triggers**	
Burns, sunburn, operation, environmental triggers (e.g., air pollution)	

^a^ References [12, 31].

SARS‐CoV‐2 infects endothelial, immune, and other cell types through the angiotensin‐converting enzyme 2 receptor. Since viral elements have been reproducibly detected in endothelial cells, infection‐associated mechanisms of cell death, such as apoptosis and pyroptosis, may explain endothelial injury in patients with coronavirus disease 2019 (COVID‐19) [33]. Multisystem inflammatory syndrome in children (MIS‐C) is a highly heterogeneous syndrome encompassing KD‐like, macrophage activation syndrome (MAS)/hemophagocytic lymphohistiocytosis (HLH)‐like, and autoimmune‐like phenotypes [34]. MIS‐C as a whole is distinct from classic KD. While histopathological data on coronary arteries in MIS‐C with KD‐like phenotype are quite limited [35, 36], the persistence of CALs in ~6% and cases of giant aneurysms [37, 38] suggest that KD‐like vascular pathology may be involved in MIS‐C with KD‐like phenotype. During the Omicron wave, 28.3% of MIS‐C cases in the United States and 72% in Japan met KD criteria [39, 40], with East Asian cases showing clinical features often indistinguishable from KD [40, 41]. CALs occurred in 11.6% and persisted in 6.2% at discharge in Japan [40], comparable to classic KD. Molecular studies indicate that MIS‐C with KD‐like phenotype shares a similar transcriptomic profile with KD [42], although immunological differences remain. For example, expansion of Vβ21.3^+^ T cells—absent in KD—has been observed in MIS‐C [43, 44], suggesting its potential as a biomarker. However, unlike classical superantigen‐mediated diseases, this expansion is limited (~3%) [43], leaving its pathogenic significance uncertain.

Regarding bacterial triggers, *Yersinia (Y) pseudotuberculosis* has been implicated in two inflammatory disorders: Far East scarlet‐like fever (FESLF) [45] and a subset of KD [46, 47, 48, 49], in addition to causing self‐limiting gastroenteritis. FESLF has been associated with *Y. pseudotuberculosis* strains carrying the *ypm* (*Y. pseudotuberculosis*‐derived mitogen) gene, and affected patients typically exhibit positive anti‐YPMa antibodies, elevated atypical lymphocytes, and activated T cells [45]. However, an increase in atypical lymphocytes (activated T cells) has not been observed in KD patients associated with *Y. pseudotuberculosis* in previous studies, including our unpublished data [46, 47, 48]. Furthermore, CALs have not been reported in cases of FESLF [45]. In Japan, 12%–35% of children infected with *Y. pseudotuberculosis* present with symptoms that fulfill the diagnostic criteria for KD [46]. In Europe, KD incidence has been observed to increase regionally and temporally with rising exposure to *Y. pseudotuberculosis* [49]. In certain regions of Japan, 9%–10% of hospitalized KD patients demonstrate serological evidence of *Y. pseudotuberculosis* infection. These patients frequently exhibit more pronounced abdominal symptoms and a higher likelihood of developing cardiac sequelae compared to KD patients without this infection [47, 48]. Similarly, *Yersinia enterocolitica* infection has been associated with KD‐like symptoms in Europe, Australia (3%, 1 out of 32 patients), the United States, and Japan (10%, 2 out of 20 patients in our observations). Most of these patients present with abdominal symptoms and incomplete KD, typically without CALs [12, 50, 51].

In summary, a subset of patients infected with *Y. pseudotuberculosis* or *Y. enterocolitica* exhibit KD symptoms, with *Yersinia*‐associated KD incidence varying by geographic region. While the *ypm* gene may play a critical role in the pathogenesis of FESLF, its role in KD is questionable [45, 52], as *Y. enterocolitica* lacks the *ypm* gene [53]. Given that *Yersinia* infection induces pyroptosis through mechanisms distinct from those activated by other microbes [54], it is plausible that pyroptosis triggered by *Yersinia* infection plays a significant role in the development of KD [12, 52].

Other bacteria, such as *Streptococcus pyogenes* and *Mycoplasma pneumoniae*, have also been linked to KD in specific regions. In Korea, KD patients with elevated anti‐streptolysin O titers experienced more severe clinical courses and higher rates of coronary artery abnormalities [55]. In China, *Mycoplasma pneumoniae* infections in older KD patients were associated with increased respiratory tract involvement [56].

Nonmicrobial triggers, including burns and severe sunburn injuries, have also been reported in association with KD. Reports from Canada, Japan, and China describe KD onset 2–5 days following burn or sunburn injuries, with 7 of the 10 patients testing negative for wound cultures [12]. All patients responded to IVIG; however, one patient developed coronary artery dilation [12]. Necrosis, the predominant form of cell death in burn injuries, releases DAMPs, contributing to monocyte and macrophage activation—a pathological hallmark in burn patients [57]. Serum levels of DAMPs, including high‐mobility group box protein 1 (HMGB1), are significantly elevated immediately after burn injuries, and in an infant with KD following severe sunburn [12, 57]. Nevertheless, the rare association of burns or sunburn injuries with KD suggests that additional host, microbial, and/or environmental factors may contribute to KD pathogenesis. Nonmicrobial environmental triggers are further discussed in the section on oxidative and nitrosative stress in KD.

### 3.2 Diversity of Incubation Periods for KD Triggers

Epidemiological studies on twins and siblings in Japan suggest that the incubation period of KD is typically within 1 week [58], aligning with the durations observed in *Yersinia pseudotuberculosis* infections, burns, and adenovirus infections [12, 32]. In contrast, many viral infections exhibit longer incubation periods before the onset of KD. For instance, KD‐like symptoms in MIS‐C typically appear approximately 1 month after SARS‐CoV‐2 infection [59]. Moreover, epidemiological studies in Korea, Chile, and the United States report a delay of about 1 month between the onset of KD and preceding seasonal infections such as respiratory syncytial virus (RSV), rhinovirus, rotavirus, norovirus, human metapneumovirus, and influenza virus infections [60, 61, 62, 63]. We experience a clear and reproducible increase in KD cases following outbreaks of infections such as RSV in Japan, where KD incidence is high. Our molecular epidemiological analysis of individual cases identified a 1‐month time lag between KD and preceding infections caused by RSV, human rhinovirus/enterovirus, and parainfluenza virus type 3 [32].

The approximate 1‐month delay between various viral infections and the onset of KD supports the hypothesis that KD is an immune‐mediated disorder rather than a direct consequence of acute infection (Table 3). In MIS‐C, it has been proposed that factors such as antibody‐dependent enhancement (ADE), autoantibodies, immune complexes, and co‐infections or superinfections with viruses or bacteria may contribute to the delayed onset of KD‐like symptoms [64]. ADE refers to a phenomenon in which non‐neutralizing or sub‐neutralizing virus‐specific antibodies, or insufficient levels of neutralizing antibodies, enhance viral entry into cells via Fc receptors or canonical viral receptors [65]. Complement‐mediated ADE may also play a role in the delayed onset of KD [64], as this process can activate intracellular innate immune inflammasome pathways, leading to the production of pro‐inflammatory cytokines and pyroptosis.

**Table 3:** Time lag between viral infections and Kawasaki disease (KD).

Country, Year [Reference]	Viral infection and time lag	Epidemiological evidence	Molecular evidence
UK, USA, Italy 2020 [24, 59]	SARS‐CoV‐2 2–6 weeks' lag to KD‐like illness	Big data	Seropositivity PCR positivity
Korea 2021 [60]	RSV, HRV, rotavirus, norovirus 1–2 months' lag	Big data	None
Korea 2022 [63]	RSV, HRV, varicella 1–3 months' lag	Big data	None
Chile 2021 [61]	RSV, influenza A, MPV 1 month's lag	Big data	None
USA 2023 [62]	RSV 2–5 weeks' lag	Big data	None
Japan 2024 [32]	RSV, HRV/EV, PIV3 1 month's lag	FCH data	Seropositivity PCR positivity

Abbreviations: EV, enterovirus; FCH, Fukuoka Children's Hospital; HRV, human rhinovirus; MPV, metapneumovirus; PCR, polymerase chain reaction; PIV3, parainfluenza virus 3; RSV, respiratory syncytial virus; SARS‐CoV‐2, severe acute respiratory syndrome coronavirus 2; UK, United Kingdom; USA, United States of America.

In severe COVID‐19, antibody‐mediated infection of monocytes by SARS‐CoV‐2 is thought to induce inflammatory cell death (pyroptosis), which triggers the release of large quantities of DAMPs [66]. These DAMPs can further propagate pyroptosis in endothelial and other cells [67], amplifying vascular inflammation and potentially contributing to the development of KD‐like disease or KD itself. However, autoantibodies alone do not fully account for the clinical features of KD, such as its age distribution, clustering, seasonality, self‐limited nature, and rare recurrence. Additionally, immune complex depositions have not been identified in KD vasculitis lesions [12].

Distinct pathogens, both viral and bacterial, activate various inflammasome pathways [68]. Co‐infections or superinfections may synergistically hyperactivate the innate immune system [69], resulting in exaggerated pyroptosis. From this perspective, pyroptosis may account for the association between KD and a broad spectrum of infectious agents. Furthermore, it provides a unifying mechanism to explain the variability in incubation periods and the role of ADE.

### 3.3 Diversity of Transmission Pathways for KD Triggers

During the COVID‐19 state of emergency, strict non‐pharmaceutical interventions (such as social distancing, mask‐wearing, and isolation) were implemented. Several studies have demonstrated that the incidence of droplet‐ and contact‐transmitted infections (RSV, rotavirus, and influenza virus) significantly decreased. However, the number of KD cases did not decline by more than half, even with the disruption of their transmission pathways [70]. This reduction pattern of KD resembled that of cutaneous and subcutaneous infections caused by community‐associated methicillin‐resistant *Staphylococcus aureus*, which are known to spread via airborne transmission [70].

An epidemiological study in France also reported that approximately 35% of KD cases could be linked to seasonal infections, suggesting that the up to 50% reduction in KD cases during the COVID‐19 pandemic may relate to decreased contact‐ or droplet‐transmitted infections [71]. Pathogens strongly associated with KD, such as *Y. pseudotuberculosis* and SARS‐CoV‐2, are primarily transmitted through droplet and/or contact routes [12]. Recent studies, however, have indicated that in older children, KD may also be associated with local or regional winds that could carry airborne environmental triggers [72]. This suggests that airborne transmission may play a role in the development of KD in a subset of cases [70, 72, 73].

## 4 Importance of DAMPs in the Presence of Microbes or PAMPs/MAMPs

The detection of microbial components in the absence of DAMPs may indicate infections with avirulent microorganisms. In contrast, the concurrent detection of microbial components and DAMPs, such as oxidized lipids, suggests encounters with actively replicating microorganisms, triggering an enhanced inflammatory and defensive immune response [74]. Pyroptosis triggered during viral or bacterial infections, as well as necrosis from burn or sunburn injuries, releases pro‐inflammatory cellular contents known as DAMPs. ROS rapidly oxidize cellular components, including membrane phospholipids in damaged cells [75]. DAMPs, such as oxidized phospholipids and low‐density lipoproteins (LDLs), subsequently activate endothelial and innate immune cells, leading to the production of pro‐inflammatory cytokines and additional ROS. These processes stimulate inflammasomes, further promoting pyroptosis in endothelial cells and monocytes [67]. DAMPs such as calreticulin, heat shock proteins, HMGB1, IL‐33, mitochondrial DNA, oxidized LDL/phospholipids, S100 proteins, and tenascin‐C are elevated in the serum of KD patients (Table 4) [12, 77, 78, 79, 80, 81].

**Table 4:** DAMPs and DAMP‐sensing receptors in Kawasaki disease.

DAMPs [Reference]	Related cell death	Serum levels in Kawasaki disease	DAMP‐sensing receptors, including PRR and SR [76]
Calreticulin	Apoptosis	Increased	SR‐L1 (CD91)
Heat shock proteins	Necroptosis NCD	Increased	TLR4, TLR2, CD91/LRP‐1, TREM1, CD14, SR‐J (RAGE), SR‐L1 (CD91)
HMGB1	Apoptosis Necroptosis Pyroptosis	Increased	TLR4, TREM1, TLR9, cGAS, AIM2, SR‐J (RAGE)
IL‐33 [77]	Necroptosis, NCD	Increased	ST2
Mitochondrial DNA [78, 79]	Necroptosis Pyroptosis	Increased	AIM2, cGAS, TLR9, IFI16, NLRP3, NLRP10, ZBP1
Oxidized LDL/phospholipids	Necroptosis Pyroptosis	Increased	TLR4, SR‐D (CD68), SR‐G (CXCL16), SR‐H (Stabilin‐1)
S100 proteins	Necroptosis NCD	Increased	TLR4, EGFR, GPCR, CD36, SR‐J (RAGE)
Tenascin‐C [80]	Necroptosis Pyroptosis	Increased	TLR4, SR‐E1 (LOX‐1)

*Note:* Updated from Hara et al. [12].

Abbreviations: AIM2, absent in melanoma 2; CD, cluster of differentiation; cGAS, cyclic GMP‐AMP synthase; CXCL16, C‐X‐C motif chemokine ligand 16; DAMP, damage‐associated molecular patterns; EGFR, epidermal growth factor receptor; GPCR, G protein‐coupled receptor; HMGB1, high‐mobility group box 1 protein; IFI16, interferon‐gamma‐inducible protein 16; IL, interleukin; LDL, low‐density lipoprotein; LOX‐1, lectin‐like oxidized low‐density lipoprotein receptor‐1; LRP‐1, low‐density lipoprotein receptor‐related protein 1; NCD, necrotic cell death; NLRP, nucleotide‐binding oligomerization domain, leucine‐rich repeat and pyrin domain containing; PRRs, pattern recognition receptors; RAGE, receptor for advanced glycation end‐products; SR, scavenger receptor; ST2, suppression of tumorigenicity 2; TLR, toll‐like receptor; TREM1, triggering receptor expressed on myeloid cells 1; ZBP1, Z‐DNA binding protein 1.

Thus, current evidence suggests that pro‐inflammatory cell death, including pyroptosis and necrosis of endothelial and innate immune cells, may play a crucial role in the development of KD vasculitis. DAMP‐sensing receptors, including PRRs and scavenger receptors, are key components of this process [76]. Further research is necessary to clarify the roles of these DAMP‐sensing receptors in KD pathogenesis.

## 5 Oxidative and Nitrosative Stress in KD

ROS and reactive nitrogen species (RNS) often exert deleterious effects on the cardiovascular system. Intracellular sources of ROS and RNS include the mitochondria, endoplasmic reticulum, lysosome, peroxisome, and certain enzymes in the cytoplasm and plasma membrane [82]. Elevated ROS levels act as concurrent messengers with inflammatory signals, activating the canonical NF‐κB pathway, inducing the expression of inflammatory and antioxidant genes, activating inflammasomes, and promoting cytokine or chemokine release [82]. Excessive production of ROS and RNS (oxidative and nitrosative stress) contributes to numerous cardiovascular pathophysiological events, including endothelial dysfunction, vascular inflammation, and atherosclerosis [82].

Inflammation can be regarded as both a cause and a consequence of increased oxidative and nitrosative stress. At active inflammation sites, inflammatory cells, vascular endothelial cells, and smooth muscle cells release ROS, RNS, enzymes, and chemical mediators, intensifying oxidative and nitrosative stress. Oxidative and nitrosative stress, in turn, stimulates the NF‐κB pathway, promoting cytokine and chemokine expression that further augments inflammation. This reciprocal relationship between oxidative and nitrosative stress and inflammation amplifies their effects [82].

**Figure 1:**
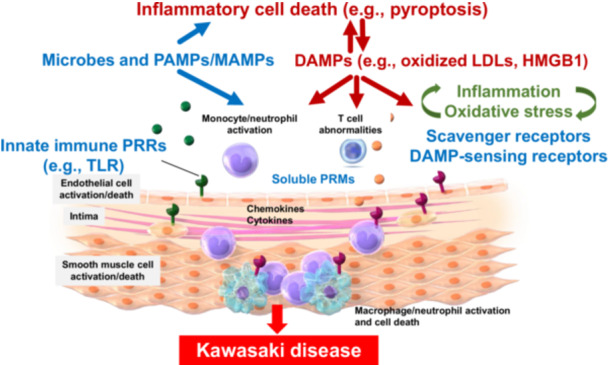
A hypothetical model for the pathophysiology of Kawasaki disease (KD). Innate immune pathogen‐associated molecular patterns (PAMPs) from microbes activate pro‐inflammatory signals in innate immune and vascular cells through pattern recognition receptors (PRRs), thereby producing large amounts of chemokines and cytokines. Massively produced damage‐associated molecular patterns (DAMPs) from cell death and oxidative stress in the circulating blood exert pleiotropic effects on platelets, monocytes, neutrophils, endothelial cells, and vascular smooth muscle cells through receptor [lectin‐like oxidized LDL receptor‐1 (LOX‐1), etc.]‐mediated and receptor‐independent mechanisms. Soluble pattern recognition molecules (PRMs) are also involved in the pathophysiology of KD. In response to these stimulations, monocytes and neutrophils in the peripheral blood are recruited to stimulated vascular cells. Subsequently, monocytes differentiate into macrophages. These macrophages and neutrophils play a pivotal role in the development of acute coronary arteritis. Inflammation and oxidative stress mutually amplify each other, leading to the induction of acute KD. HMGB1, high‐mobility group box 1; LDL, low‐density lipoprotein; MAMPs, microbe‐associated molecular patterns.

During the acute phase of KD (Figure 1), neutrophils and macrophages, the primary ROS sources, heavily infiltrate the coronary arteries [19]. ROS extend damage to inflammatory cells and adjacent vascular cells [83]. Activated neutrophils and monocytes release large amounts of myeloperoxidase, a pro‐oxidant enzyme that amplifies ROS production and contributes to the development of coronary arteritis in KD. Additionally, plasma levels of nitrogen oxides (Nox) and NO‐derived species (3‐nitrotyrosine), inducible *NOS* mRNA levels in mononuclear cells, and amounts of NO produced by neutrophils are elevated in patients with acute KD [12, 83]. Thus, oxidative and nitrative stress are simultaneously elevated during acute KD [12, 83].

Secondary ROS products also contribute to the development of vascular diseases. Lipid peroxidation products, such as oxidized phospholipids, play roles in regulating NF‐κB activation, inflammation, thrombosis, angiogenesis, endothelial function, and immune tolerance [82, 84]. Of note, oxidized phospholipids or LDLs activate the innate immune system, promoting the production of various pro‐inflammatory molecules—cytokines, chemokines, eicosanoids, proteases, ROS, and RNS—leading to immune cell recruitment and arterial wall inflammation [82]. In addition to these pro‐inflammatory functions, oxidized phospholipids exert pro‐thrombotic effects on a variety of cell types in the vessel walls [82].

During the acute phase of KD, an excessive amount of oxidative stress‐associated molecules is generated as inflammation by‐products. Indeed, lipid peroxidation products, including malondialdehyde, F2‐isoprostanes, and oxidized phospholipids and LDLs, are elevated [12, 28, 85]. Oxidized phospholipids induce arterial wall inflammation in humans [86], and oxidized LDLs cause pyroptosis in vascular endothelial cells [87]. Thus, blood levels of oxidized phosphatidylcholines and oxidized LDLs have been associated with the development of coronary arteritis [28, 85].

Lectin‐like oxidized LDL receptor‐1 (LOX‐1) is a major scavenger receptor for oxidized LDLs in vascular cells [88] and is involved in endothelial dysfunction, smooth muscle cell migration and proliferation, inflammation, and atherogenesis [89]. The LOX‐1 ligand assay measures the biological activity of apolipoprotein B based on its binding to LOX‐1 [89]. Using immobilized recombinant human LOX‐1, various types of modified LDLs can be detected as LOX‐1 ligands in patients with KD [28]. Therefore, the LOX‐1 ligand assay may more accurately reflect the pathogenic activities of apolipoprotein B than traditional oxidized lipid or oxidized LDL measurements by LC‐MS or enzyme‐linked immunosorbent assays using monoclonal antibodies. This assay could be valuable in the diagnosis of acute KD.

Spatiotemporal analyses have consistently demonstrated a positive association between long‐term exposure to particulate matter (PM) 2.5 and KD incidence in children [90, 91]. Moreover, systemic oxidative stress, indicated by elevated blood diacron‑reactive oxygen metabolite (d‐ROM) levels, appears to play a complementary role in the pathogenesis of KD, alongside inflammation [92].

## 6 Universal Genetic Backgrounds Common to KD Patients of Diverse Ethnicities

Three KD‐associated genes—*Inositol‐triphosphate 3‐kinase C* (*ITPKC*), *Caspase 3* (*CASP3*), and *Fcγ receptor IIa* (*FCGR2A*)—have been validated as KD susceptibility genes in independent cohorts across diverse ethnic groups [93, 94, 95, 96, 97, 98]. The KD‐associated *ITPKC* polymorphism has been linked to reduced mRNA production. Initially, it was hypothesized that the mutant allele might contribute to the development of KD by intensifying T‐cell‐mediated immune responses, given that ITPKC is a negative regulator of T cells [93]. However, using an ITPKC‐deficient mouse model, a mechanistic link for the *ITPKC* gene to innate immunity has been established [99]. In this model, ITPKC‐deficient macrophages exhibited increased calcium flux upon stimulation, enhanced NLRP3 inflammasome activation, and elevated IL‐1β production. This KD model, in which coronary arteritis is induced by LCWE, aligns with findings in KD patients: individuals with the high‐risk *ITPKC* polymorphism showed decreased ITPKC protein expression, increased calcium flux, and upregulated NLRP3 expression, resulting in heightened inflammasome activation [99].

Caspase 3 is an executioner caspase that plays a critical role in apoptosis. The G‐to‐A variant in exon 1 of *CASP3* (rs113420705) attenuates the DNA‐binding affinity of the nuclear factor of activated T cells (NFAT), thereby decreasing *CASP3* mRNA transcription [93]. It was considered that this *CASP3* variant may result in less immune cell apoptosis and exaggerated immune reactions [93]. Alternatively, in the presence of bacterial toxins, reduced activation of caspase 3 may lead to decreased inactivation of gasdermin D, thereby increasing pyroptosis [100]—a process closely associated with the pathophysiology of KD [101].

Regarding the *FCGR2A* gene, the His131 variant of human FcγRIIa binds human IgG2 complexes with high affinity, whereas the Arg131 variant has poor IgG2 binding. Individuals carrying the FcγRIIa‐His131 allele are generally more resistant to infections, likely due to more efficient IgG2 binding and enhanced effector responses, such as phagocytosis [102]. However, while the FCGR2A‐H131 genetic variant offers protection against infections, it is also associated with an increased risk of developing KD [102], possibly due to its enhanced activation of the innate immune system. Additionally, Fcγ receptors, particularly FcγRIIa, are essential in mediating ADE of viral infections under specific conditions, such as narrow IgG concentration ranges, particular cell types, and the presence or absence of complement [103]. In ADE, non‐neutralizing or sub‐neutralizing antibodies form complexes with viruses and bind to Fcγ receptors on immune cells, promoting viral entry, replication, and subsequent inflammasome activation and pyroptosis. This mechanism is particularly relevant for viruses like dengue virus, influenza virus, and SARS‐CoV‐2 [66, 104]. The H131 genotype, with its increased FcγRIIa expression, may exacerbate this phenomenon, potentially leading to more severe disease outcomes [104].

These genetic variants alone are insufficient to account for the heightened susceptibility to KD observed in Asian populations [96]. The low concordance rates for KD in identical twins (14%–25%) [105, 106] further suggest that environmental and other nongenetic factors play a more critical role in the development of KD among individuals of the same ethnicity.

## 7 Initial and Subsequent Immune Reactions to the Development of KD

Initial triggers for KD include both microbial (viral, bacterial, and fungal pathogens) and nonmicrobial factors. The initial immune responses in KD consist of molecular pattern‐ and antigen‐driven activation of the innate and acquired immune system, followed by a reactive innate immune phase. Under normal physiological conditions, activation of the innate immune system is stringently regulated to prevent excessive systemic inflammation and tissue damage. In contrast, the acute phase of KD is characterized by pronounced innate immune hyperactivation, accompanied by a Th17‐skewed immune response and a concomitant suppression of most T and B cell functions [9, 22, 107].

A similar condition has been observed with SARS‐CoV‐2 infection. The virus (trigger) initially enters the host cells as a conventional virus. In certain individuals, the cytopathic effects of SARS‐CoV‐2 can subsequently induce apoptosis and pyroptosis in endothelial and immune cells [33, 66]. In patients with severe COVID‐19 and MIS‐C, DAMPs, such as HMGB1 and S100A, are significantly elevated [108]. This suggests that DAMPs released during pyroptosis may further activate the innate immune system after infection [109, 110]. Such immune activation could potentially contribute to the onset of KD symptoms in children exposed to SARS‐CoV‐2.

The acute phase of KD is primarily characterized by the hyperactivation of innate immunity [22]. This concept is further supported by a recent study of the surface expression of CD14 on monocytes [92]. CD14 serves as a coreceptor of PRRs and binds to a broad spectrum of PAMPs/MAMPs and DAMPs. The CD14‐TLR‐ligand complex is internalized, resulting in a decrease of CD14 expression on the monocyte surface and innate immune activation. During the acute phase, most KD patients showed a prominent CD14 down‐modulation on monocytes, reflecting the presence of circulating innate immune molecular patterns, PAMPs/MAMPs, and DAMPs. Notably, the surface expression of CD14 on monocytes was restored concurrently with responses to IVIG and infliximab treatment in KD [92]. These data further support the importance of PAMPs/MAMPs and DAMPs in the pathophysiology of acute KD.

Furthermore, the pathogenesis of KD appears to be closely related to pyroptosis through the canonical pathway (caspase 1, inflammasomes, and gasdermin D), the noncanonical pathway (caspases 4 and 5, and gasdermin D), and potentially the apoptotic caspase‐mediated pathway (caspases 8 and 3, and gasdermins D, C, and B). Such inflammatory cell death of endothelial cells is associated with the development of vasculitis in KD [101, 111, 112, 113]. Elevated serum levels of NLRP3 have been reported in KD patients, with significantly higher levels observed in those with coronary artery aneurysms. These findings suggest a pivotal role of NLRP3, a key component of the NLRP3 inflammasome, in the pathogenesis of KD and the formation of coronary artery aneurysms [114].

Patients with KD have the highest recurrence rate within 12 months following the first episode, and this high‐incidence rate of recurrence has been observed in patients who showed cardiac sequelae during the first episode [115]. In addition, patients with a recurrent episode of KD are more likely to have coronary artery abnormalities than those without recurrence [116]. A short interval between onset and recurrence, as well as recurrence in a more severe form than the initial symptoms in KD, suggests the involvement of innate immune memory (trained immunity) [117]. Trained immunity represents the condition in which innate immune cells can undergo metabolic and epigenetic reprogramming, resulting in enhanced immune responses to heterologous reinfection or endogenous danger signals [117]. This concept may also be applicable to the cells of nonhematopoietic origin, such as epithelial cells, endothelial cells, and vascular smooth muscle cells [118]. In fact, endothelial cells function as sentinel innate immune cells and detect foreign pathogens and endogenous danger signals in the bloodstream [118]. Oxidized phospholipids and LDLs, as DAMPs, modify the epigenetic status of monocytes and vascular cells, facilitate memory responses, and boost inflammation [119]. Since oxidized phospholipids and LDLs are elevated during the acute phase of KD [28], we hypothesize that such DAMPs may reprogram the cellular metabolism and boost hyperinflammation and inflammatory cell death (pyroptosis) of innate immune cells and vascular cells in patients with KD during its onset and recurrence (Figure 1).

## 8 Involvement of the Acquired Immune System: The Role of Anti‐DAMP Antibodies

KD is also regarded as a condition that is associated with acquired immune abnormalities. The elevated blood levels of oxidized phospholipids and LDLs observed in patients with acute KD [28] may contribute to T cell anergy while simultaneously activating innate immune cells and endothelial cells [84]. Oxidized LDLs lead to a significant elevation of Th17 cells and a reduction of regulatory T cells in a dose‐ and time‐dependent manner [84]. A recent study identified elevated levels of IL‐17A, IL‐17C, and IL‐17F as hallmarks of KD [120]. While IL‐17A and IL‐17F are produced by both Th17 cells and innate immune cells, IL‐17C is predominantly secreted by epithelial cells [121]. This further suggests that the involvement of innate immune cells may represent a unique pathophysiological feature of KD.

The age‐restricted occurrence of KD, rarely seen before 6 months or after 5 years of age, suggests an involvement of maternal (transplacental) antibodies or self‐produced antibodies in protective immunity against KD‐associated pathogens [122]. In addition to antibodies against pathogenic microbes, PAMPs or MAMPs, anti‐DAMP antibodies, particularly those targeting oxidized LDL, have been identified in healthy children, age‐matched individuals with febrile illnesses, and KD patients [123]. The ability of anti‐DAMP antibodies to prevent vasculitis in KD murine models [81] suggests that these antibodies may also confer protective effects against KD‐associated vasculitis in humans from early childhood, irrespective of the trigger.

**Figure 2:**
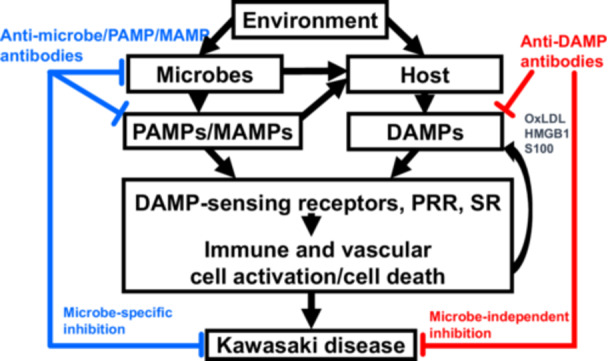
Roles of anti‐DAMP antibodies in the development of KD vasculitis. PAMPs induce the production and release of DAMP, such as oxidized LDL, HMGB1, and S100 proteins from host cells. These molecules activate immune and vascular cells cooperatively through innate immune PRRs, DAMP‐sensing receptors, and SR. Recruitment of immune cells to activated endothelial cells and destruction of vascular structures result in the development of KD vasculitis and aneurysms in genetically predisposed individuals. Anti‐microbe/PAMP‐specific antibodies neutralize their corresponding molecules, thereby inhibiting the development of KD. Similarly, if anti‐DAMP antibodies neutralize DAMPs, they can prevent the onset of KD vasculitis, regardless of the trigger, as demonstrated in murine KD models [81]. DAMP, damage‐associated molecular pattern; HMGB1, high mobility group box 1; KD, Kawasaki disease; MAMPs, microbe‐associated molecular patterns; oxLDL, oxidized low‐density lipoprotein; PAMPs, pathogen‐associated molecular patterns; PRRs, pattern recognition receptors; SR, scavenger receptors.

Our recent study demonstrated that IVIG administration significantly elevated anti‐oxidized LDL antibody levels in KD patients and concurrently improved KD symptoms, suggesting a protective role of anti‐oxidized LDL antibodies against vascular inflammation [124]. This is consistent with previous findings showing the presence of anti‐oxidized LDL antibodies in IVIG preparations [125]. The low recurrence rate of KD, ranging from 1.5% in Canada to 3%–4% in Japan [126], argues against a multiple‐etiology theory for KD, if KD triggers work via antigen‐driven mechanisms. Instead, the infrequency of KD recurrence (1%–4%) may be attributed to the presence of anti‐DAMP antibodies, which could suppress KD development regardless of the microbial trigger (Figure 2), as demonstrated in murine models [81].

The absence of documented KD cases before 1950 in Japan may be explained, at least in part, by the production of anti‐PAMP/MAMPs and anti‐DAMP antibodies from various infections. Conversely, the rise in KD cases up until the COVID‐19 pandemic might be explained by the reduction of infectious diseases and the decreased production of antimicrobial and anti‐DAMP antibodies, through the improvements in sanitation, the widespread use of antibiotics, and vaccination programs.

Notably, nationwide epidemiological data from Japan revealed a significant decline in the incidence of KD among infants aged 3–5 months following the introduction of routine PCV immunization in 2013 [127]. In England, the risk of KD onset was also markedly reduced (incidence rate 0.30) within 4 weeks of PCV administration, particularly when co‐administered with the measles, mumps, and rubella vaccine [128]. These observations may be partly explained by emerging evidence that pneumococcal vaccination induces cross‐reactive anti‐oxidized LDL antibodies. This is likely attributable to the presence of phosphorylcholine in the *Streptococcus pneumoniae* cell wall, which structurally mimics oxidized LDL [129]. Indeed, PCV13 vaccination has been associated with increased anti‐oxidized LDL antibody titers in adults [130]. Further investigation is warranted to elucidate the relationship among PCV immunization, anti‐oxidized LDL antibody production, and the reduced incidence of KD.

## 9 The Diagnosis and Treatment of KD From a Pathophysiological Standpoint

The diagnosis of KD is based on clinical criteria, with modifications from the initial one (1970), and no pathognomonic laboratory tests currently exist [2, 131]. From a pathophysiological standpoint, at least four categories of biomarkers are useful in aiding the diagnosis of KD: (a) inflammatory markers such as white blood cell count, C‐reactive protein (CRP), and procalcitonin [2]; (b) innate immune markers, including direct measurement of serum levels of specific DAMPs such as oxidized LDL, HMGB1, and S100 proteins [12, 28] or flow cytometric detection of CD14 downregulation, indicative of the presence of circulating DAMPs/PAMPs/MAMPs [92]; (c) oxidative stress markers such as reactive oxygen metabolites‐derived compounds (d‐ROMs) and biological antioxidant potential [92]; and (d) cardiovascular markers, notably N‐terminal prohormone of brain natriuretic peptide [2]. Real‐time assessment of CD14 modulation on monocytes could be a valuable strategy for evaluating treatment response, supporting differential diagnosis, and guiding the selection of second‐ or third‐line therapies following IVIG administration in patients with KD [92].

Five immunomodulatory therapies commonly employed in the management of KD are discussed from a pathogenic and pathophysiological perspective.

1. IVIG:
  IVIG reduces systemic inflammation and protects against coronary artery damage through multiple potential mechanisms. These include the neutralization of microbial components (PAMPs/MAMPs), toxins, antigens, and cytokines; blockade of Fc receptors; modulation of cytokine production; and stimulation of regulatory T cells [132]. Notably, the therapeutic efficacy of IVIG in KD is substantially greater than in other vasculitic disorders [133], suggesting that distinct or additional mechanisms may be involved. Microbial proteins such as Protein A (from *Staphylococcus aureus*), Protein G (from group G/C streptococci), and Protein L (from *Peptostreptococcus magnus*) are well known to bind immunoglobulins independently of their antigen‐binding domains [134]. Our previous work demonstrated that PAMPs/MAMPs and DAMPs derived from KD patients can bind directly to the constant region of IgG—outside the variable domain—as shown using IgG affinity column experiments [135]. Furthermore, Ficolin‐M, a soluble pattern recognition molecule of the innate immune system, was identified as the most significantly decreased protein following IVIG therapy in KD patients [136]. Its collagen‐like domain binds to the constant region (CH1–CH3 domains) of IgG rather than the variable region, suggesting a mechanism by which KD‐associated PAMPs/MAMPs and DAMPs may be cleared. Additionally, anti‐DAMP antibodies, such as anti‐oxidized LDL antibodies present in IVIG preparations, may also contribute to the therapeutic benefit [123, 125].
2. Corticosteroids:
  Corticosteroids (e.g., prednisolone) pass through cell membranes and bind to cytoplasmic glucocorticoid receptors (GRs), which are ubiquitously expressed. The steroid‐GR complex then translocates to the nucleus, where it modulates gene expression at the transcriptional level. This interaction suppresses key inflammatory pathways, leading to reduced production of pro‐inflammatory cytokines such as IL‐1β and TNF‐α [137]. In vitro studies using human coronary artery endothelial cells have shown that corticosteroids inhibit extracellular HMGB1 release, a representative DAMP [138]. However, in our study, administration of 2 mg/kg of corticosteroids initiated after the second IVIG infusion did not significantly reduce DAMP and PAMP/MAMPs levels after 3 days of oral treatment [92], possibly due to differences in cellular responses between in vitro and in vivo models, disease severity, or timing of administration.
3. TNF‐α antagonists (monoclonal antibody: Infliximab; soluble receptor: Etanercept):
  Infliximab is a monoclonal antibody that binds with high affinity to both soluble and transmembrane TNF‐α, thereby disrupting pro‐inflammatory signaling cascades. It suppresses cytokine production (e.g., IL‐1 and IL‐6), inhibits leukocyte trafficking, and induces apoptosis in TNF‐producing cells such as activated monocytes/macrophages, neutrophils, and vascular smooth muscle cells [139]. In our study, infliximab given after two IVIG courses led to a significant reduction in DAMPs and PAMPs/MAMPs [92], possibly through apoptotic elimination of activated monocytes. In contrast, etanercept, a soluble TNF receptor, does not induce cell apoptosis but binds and neutralizes both TNF‐α and TNF‐β [131].
4. Cyclosporine:
  Initially recognized as a T‐cell‐specific inhibitor via blockade of the calcium‐calcineurin–NFAT pathway [131], cyclosporine is now known to also affect macrophages, dendritic cells, and B cells, and is used in the treatment of macrophage activation syndrome [140]. In the acute phase of KD, monocyte activation exceeds T‐cell activation in peripheral blood, and histopathology reveals widespread macrophage/monocyte infiltration in vasculitic lesions [22]. This suggests that cyclosporine may exert therapeutic effects primarily through modulation of macrophages/monocytes rather than T cells [141, 142].
5. Plasma Exchange:
  Plasma exchange has proven effective and safe for treating CALs in KD patients refractory to IVIG [143]. Its therapeutic benefits are likely due to the removal of inflammatory mediators, such as TNF‐α, IL‐1β, IL‐6, IL‐17, and granulocyte colony stimulating factor [144], as well as the potential clearance of circulating DAMPs and PAMPs/MAMPs.

## 10 Future Directions for Elucidating the Etiopathogenesis of KD

Current evidence supports a multifactorial etiology of KD, wherein genetically predisposed individuals develop KD in response to diverse environmental and/or microbial stimuli. The difficulty in elucidating the etiopathogenesis of KD arises from several interrelated factors: (a) The molecular diversity of PAMPs, MAMPs, and DAMPs—particularly oxidized LDL—which exist in highly heterogeneous forms [28]. The specific oxidized LDL molecular species associated with KD onset or the development of CAL remain unidentified; (b) Antibodies targeting PAMPs, MAMPs, and DAMPs, including various oxidized LDL species, are themselves highly heterogeneous. Current assays measure total anti‐oxidized LDL antibody levels without differentiating potentially protective subsets that may confer resistance to KD onset. Additionally, the abundance of anti‐PAMP/MAMP/DAMP antibodies, which may play a protective role, likely varies depending on previous infections and host immune status; (c) Environmental factors, including markers of oxidative stress (e.g., d‐ROMs), may further contribute to KD onset. Given the potential complementary role of inflammation and oxidative stress in KD pathogenesis [92], these parameters must be evaluated concurrently. Together, these complex and interacting factors underscore the multifactorial nature of KD. The interplay among diverse environmental triggers, inflammatory mediators, components of the innate and adaptive immune systems, oxidative stress, and their corresponding regulatory antibodies has made it difficult to reach a definitive etiological conclusion.

## 11 Conclusions

Current evidence indicates that KD develops in genetically predisposed individuals upon exposure to a microbial and/or nonmicrobial trigger(s), which may vary across geographic regions and environmental conditions. As shown in Table 5, various etiologic hypotheses appeared since the 1960s with the identification of genetic and environmental factors. At present, both microbes/PAMPs/MAMPs and inflammatory cell death‐associated DAMPs appear to play critical roles in KD pathogenesis. The interplay between inflammation and oxidative stress further amplifies immune responses, potentially contributing to KD onset. The presence of anti‐DAMP antibodies may provide insight into the relatively low recurrence rate of KD, despite the diverse range of KD‐inducing agents. Future research is expected to elucidate the intricate immune mechanisms, environmental factors, and genetic predispositions underlying KD, paving the way for evolving diagnostic, therapeutic, and preventive strategies.

**Table 5:** Time sequence of etiologic hypotheses for Kawasaki disease (KD).

Time period	Etiologic hypothesis	
1960s~	Infectious disease hypothesis	A wide range of pathogens—including viruses, bacteria, fungi, and their components—were proposed as potential triggers. Some were reproducible [12, 31].
1992~	Superantigen hypothesis	Superantigens derived from *Staphylococcus aureus* were proposed to be linked to KD pathogenesis [145]. However, the hypothesis lacked consistent confirmation.
2004~	New RNA virus hypothesis	Rowley AH and colleagues proposed the involvement of a novel RNA virus [146, 147, 148]. Despite efforts, a specific virus has not been identified.
2008~	Identification of universal genetic factors	Genome‐wide association studies identified *ITPKC*, *CASP3*, and *FCGR2A* as universal susceptibility genes [93, 94, 95, 96, 97, 98].
2011~	PAMPs/MAMPs hypothesis	A novel murine model of KD was established using a Nod1 ligand, suggesting the role of PAMPs/MAMPs in initiating coronary arteritis [8].
2016~	PAMPs/MAMPs and DAMPs hypothesis	Emerging data support the role of DAMPs, such as oxidized LDLs, in conjunction with PAMPs/MAMPs. Elevated oxidized LDL levels are linked to CAL formation [12, 22, 28, 85].
2017~	Environmental factors	Recent studies indicate that exposure to air pollutants may act as amplifiers in KD development [90, 91, 149].
2020~	Emergence of MIS‐C	Research on MIS‐C cases with KD‐like features has contributed to a deeper understanding of KD pathogenesis [42].
2024~	Immune and environmental triggers hypothesis	Complementary effects of PAMPs/MAMPs/DAMPs and environmental triggers on individuals with genetic susceptibility lead to KD development [92].

Abbreviations: CAL, coronary artery lesion; DAMPs, damage‐associated molecular patterns; LDLs, low‐density lipoproteins; MAMPs, microbe‐associated molecular patterns; MIS‐C, multisystem inflammatory syndrome in children; PAMPs, pathogen‐associated molecular patterns.

## Author Contributions

**Toshiro Hara:** conceptualization, writing – original draft, writing – review and editing, visualization, resources, supervision. **Yasunari Sakai:** conceptualization, writing – original draft, writing – review and editing, funding acquisition, resources.

## Conflicts of Interest

The authors declare no conflicts of interest.

## Data Availability

The review article does not contain the original data in the manuscript.

## References

[1] D. Burgner and A. Harnden , “Kawasaki Disease: What Is the Epidemiology Telling us About the Etiology?,” International Journal of Infectious Diseases 9 (2005): 185–194. 15936970 10.1016/j.ijid.2005.03.002 PMC7110839

[2] R. Fukazawa , J. Kobayashi , M. Ayusawa , et al., “JCS/JSCS 2020 Guideline on Diagnosis and Management of Cardiovascular Sequelae in Kawasaki Disease,” Circulation Journal 84 (2020): 1348–1407. 32641591 10.1253/circj.CJ-19-1094

[3] J. C. Burns , H. I. Kushner , J. F. Bastian , et al., “Kawasaki Disease: A Brief History,” Pediatrics 106 (2000): e27. 10920183 10.1542/peds.106.2.e27

[4] C. Manlhiot , B. Mueller , S. O'Shea , et al., “Environmental Epidemiology of Kawasaki Disease: Linking Disease Etiology, Pathogenesis and Global Distribution,” PLoS One 13 (2018): e0191087. 29415012 10.1371/journal.pone.0191087 PMC5802431

[5] D. van Stijn , A. Slegers , H. Zaaijer , and T. Kuijpers , “Corrigendum: Lower CMV and EBV Exposure in Children With Kawasaki Disease Suggests an Under‐Challenged Immune System,” Frontiers in Pediatrics 9 (2021): 765546. 34568246 10.3389/fped.2021.765546 PMC8461748

[6] N. Nagi‐Miura , Y. Adachi , and N. Ohno , “Coronary Arteritis Induced by CAWS (*Candida albicans* Water‐Soluble Fraction) in Various Strains of Mice,” Nihon Ishinkin Gakkai Zasshi 49 (2008): 287–292. 19001755 10.3314/jjmm.49.287

[7] T. J. A. Lehman , R. Warren , D. Gietl , V. Mahnovski , and M. Prescott , “Variable Expression of *Lactobacillus casei* Cell Wall‐Induced Coronary Arteritis: An Animal Model of Kawasaki's Disease in Selected Inbred Mouse Strains,” Clinical Immunology and Immunopathology 48 (1988): 108–118. 3133145 10.1016/0090-1229(88)90161-4

[8] H. Nishio , S. Kanno , S. Onoyama , et al., “Nod1 Ligands Induce Site‐Specific Vascular Inflammation,” Arteriosclerosis, Thrombosis, and Vascular Biology 31 (2011): 1093–1099. 21330608 10.1161/ATVBAHA.110.216325

[9] Y. Motoji , R. Fukazawa , R. Matsui , et al., “Kawasaki Disease‐Like Vasculitis Facilitates Atherosclerosis, and Statin Shows a Significant Antiatherosclerosis and Anti‐Inflammatory Effect in a Kawasaki Disease Model Mouse,” Biomedicines 10 (2022): 1794. 35892695 10.3390/biomedicines10081794 PMC9330289

[10] S. Chen , Y. Lee , T. R. Crother , et al., “Marked Acceleration of Atherosclerosis After *Lactobacillus casei*‐Induced Coronary Arteritis in a Mouse Model of Kawasaki Disease,” Arteriosclerosis, Thrombosis, and Vascular Biology 32 (2012): e60‐71. 22628430 10.1161/ATVBAHA.112.249417 PMC3480341

[11] S. Kanno , H. Nishio , T. Tanaka , et al., “Activation of an Innate Immune Receptor, Nod1, Accelerates Atherogenesis in Apoe‐/‐ Mice,” Journal of Immunology 194 (2015): 773–780. 10.4049/jimmunol.1302841 25488987

[12] T. Hara , K. Yamamura , and Y. Sakai , “The Up‐to‐Date Pathophysiology of Kawasaki Disease,” Clinical & Translational Immunology 10 (2021): e1284. 33981434 10.1002/cti2.1284 PMC8109476

[13] A. T. Stock , J. A. Hansen , M. A. Sleeman , B. S. McKenzie , and I. P. Wicks , “GM‐CSF Primes Cardiac Inflammation in a Mouse Model of Kawasaki Disease,” Journal of Experimental Medicine 213 (2016): 1983–1998. 27595596 10.1084/jem.20151853 PMC5030799

[14] F. Anzai , S. Watanabe , H. Kimura , et al., “Crucial Role of NLRP3 Inflammasome in a Murine Model of Kawasaki Disease,” Journal of Molecular and Cellular Cardiology 138 (2020): 185–196. 31836541 10.1016/j.yjmcc.2019.11.158

[15] R. Tada , Y. Takano , H. Murakami , et al., “Vasculitis and Anaphylactoid Shock in Mice Induced by the Polysaccharide Fraction Secreted Into Culture Supernatants by the Fungus *Candida metapsilosis* ,” Microbiology and Immunology 55 (2011): 357–365. 21362025 10.1111/j.1348-0421.2011.00326.x

[16] Y. Lee , D. Wakita , J. Dagvadorj , et al., “IL‐1 Signaling Is Critically Required in Stromal Cells in Kawasaki Disease Vasculitis Mouse Model: Role of Both IL‐1α and IL‐1β,” Arteriosclerosis, Thrombosis, and Vascular Biology 35 (2015): 2605–2616. 26515418 10.1161/ATVBAHA.115.306475 PMC4662607

[17] O. Adewuya , Y. Irie , K. Bian , E. Onigu‐Otite , and F. Murad , “Mechanism of Vasculitis and Aneurysms in Kawasaki Disease: Role of Nitric Oxide,” Nitric oxide 8 (2003): 15–25. 12586537 10.1016/s1089-8603(02)00125-8

[18] D. J. Schulte , A. Yilmaz , K. Shimada , et al., “Involvement of Innate and Adaptive Immunity in a Murine Model of Coronary Arteritis Mimicking Kawasaki Disease,” Journal of Immunology 183 (2009): 5311–5318. 10.4049/jimmunol.0901395 PMC3031986 19786535

[19] K. Takahashi , T. Oharaseki , S. Naoe , M. Wakayama , and Y. Yokouchi , “Neutrophilic Involvement in the Damage to Coronary Arteries in Acute Stage of Kawasaki Disease,” Pediatrics International 47 (2005): 305–310. 15910456 10.1111/j.1442-200x.2005.02049.x

[20] R. Ohashi , R. Fukazawa , M. Watanabe , et al., “Characterization of a Murine Model With Arteritis Induced by Nod1 Ligand, FK565: A Comparative Study With a CAWS‐Induced Model,” Modern Rheumatology 27 (2017): 1024–1030. 28150515 10.1080/14397595.2017.1287150

[21] Y. Motomura , S. Kanno , K. Asano , et al., “Identification of Pathogenic Cardiac CD11c+ Macrophages in Nod1‐Mediated Acute Coronary Arteritis,” Arteriosclerosis, Thrombosis, and Vascular Biology 35 (2015): 1423–1433. 25838430 10.1161/ATVBAHA.114.304846

[22] T. Hara , Y. Nakashima , Y. Sakai , H. Nishio , Y. Motomura , and S. Yamasaki , “Kawasaki Disease: A Matter of Innate Immunity,” Clinical and Experimental Immunology 186 (2016): 134–143. 27342882 10.1111/cei.12832 PMC5054572

[23] D. P. Burgner , M. N. Cooper , H. C. Moore , et al., “Childhood Hospitalisation With Infection and Cardiovascular Disease in Early‐Mid Adulthood: A Longitudinal Population‐Based Study,” PLoS One 10 (2015): e0125342. 25938548 10.1371/journal.pone.0125342 PMC4418819

[24] L. Verdoni , A. Mazza , A. Gervasoni , et al., “An Outbreak of Severe Kawasaki‐Like Disease at the Italian Epicentre of the SARS‐CoV‐2 Epidemic: An Observational Cohort Study,” Lancet 395 (2020): 1771–1778. 32410760 10.1016/S0140-6736(20)31103-X PMC7220177

[25] R. S. Yeung , “Phenotype and Coronary Outcome in Kawasaki's Disease,” Lancet 369 (2007): 85–87. 17223454 10.1016/S0140-6736(07)60045-2

[26] S. Kido , R. Ae , K. Kosami , et al., “Seasonality of i.v. Immunoglobulin Responsiveness in Kawasaki Disease,” Pediatrics International 61 (2019): 539–543. 30980447 10.1111/ped.13863

[27] D. Shimizu , T. Hoshina , M. Kawamura , et al., “The Possible Association Between Epidemics of Hand‐Foot‐and‐Mouth Disease and Responsiveness to Immunoglobulin Therapy in Kawasaki Disease,” Frontiers in Pediatrics 10 (2022): 968857. 36147800 10.3389/fped.2022.968857 PMC9485717

[28] Y. Nakashima , Y. Sakai , Y. Mizuno , et al., “Lipidomics Links Oxidized Phosphatidylcholines and Coronary Arteritis in Kawasaki Disease,” Cardiovascular Research 117 (2021): 96–108. 31782770 10.1093/cvr/cvz305

[29] Y. Torii , K. Horiba , S. Hayano , et al., “Comprehensive Pathogen Detection in Sera of Kawasaki Disease Patients by High‐Throughput Sequencing: A Retrospective Exploratory Study,” BMC Pediatrics 20 (2020): 482. 33059644 10.1186/s12887-020-02380-7 PMC7557310

[30] H. Jackson , S. Menikou , S. Hamilton , et al., “Kawasaki Disease Patient Stratification and Pathway Analysis Based on Host Transcriptomic and Proteomic Profiles,” International Journal of Molecular Sciences 22 (2021): 5655. 34073389 10.3390/ijms22115655 PMC8198135

[31] W. Wang , L. Zhu , X. Li , Z. Liu , H. Lv , and G. Qian , “Emerging Evidence of Microbial Infection in Causing Systematic Immune Vasculitis in Kawasaki Disease,” Frontiers in Microbiology 14 (2023): 1313838. 38188572 10.3389/fmicb.2023.1313838 PMC10771848

[32] K. Marutani , K. Murata , Y. Mizuno , et al., “Respiratory Viral Infections and Kawasaki Disease: A Molecular Epidemiological Analysis,” Journal of Microbiology, Immunology and Infection 57 (2024): 691–699. 10.1016/j.jmii.2024.07.001 39034166

[33] Z. Varga , A. J. Flammer , P. Steiger , et al., “Endothelial Cell Infection and Endotheliitis in COVID‐19,” Lancet 395 (2020): 1417–1418. 32325026 10.1016/S0140-6736(20)30937-5 PMC7172722

[34] M. Ramos‐Casals , P. Brito‐Zerón , and X. Mariette , “Systemic and Organ‐Specific Immune‐Related Manifestations of COVID‐19,” Nature Reviews Rheumatology 17 (2021): 315–332. 33903743 10.1038/s41584-021-00608-z PMC8072739

[35] J. Mayordomo‐Colunga , A. Vivanco‐Allende , I. López‐Alonso , et al., “SARS‐CoV‐2 Spike Protein in Intestinal Cells of a Patient With Coronavirus Disease 2019 Multisystem Inflammatory Syndrome,” Journal of Pediatrics 243 (2022): 214–218.e5. 34843710 10.1016/j.jpeds.2021.11.058 PMC8626144

[36] A. N. Duarte‐Neto , E. G. Caldini , M. S. Gomes‐Gouvêa , et al., “An Autopsy Study of the Spectrum of Severe COVID‐19 in Children: From SARS to Different Phenotypes of MIS‐C,” EClinicalMedicine 35 (2021): 100850. 33937731 10.1016/j.eclinm.2021.100850 PMC8072136

[37] A. Baykan , Y. E. Kum , M. M. Yılmazer , et al., “One‐Year Follow‐Up Results of MIS‐C Patients With Coronary Artery Involvement: A Multi‐Center Study,” Pediatric Cardiology 45 (2024): 282–291. 38159144 10.1007/s00246-023-03364-x

[38] D. S. Villacis‐Nunez , S. Hashemi , M. C. Nelson , et al., “Giant Coronary Aneurysms in Multisystem Inflammatory Syndrome in Children Associated With SARS‐CoV‐2 Infection,” JACC: Case Reports 3 (2021): 1499–1508. 34642670 10.1016/j.jaccas.2021.06.043 PMC8494056

[39] B. W. McCrindle , A. S. Harahsheh , R. Handoko , et al., “SARS‐CoV‐2 Variants and Multisystem Inflammatory Syndrome in Children,” New England Journal of Medicine 388 (2023): 1624–1626. 36947454 10.1056/NEJMc2215074 PMC10052214

[40] D. Matsubara , Y. Matsubara , M. Ayusawa , et al., “Nationwide Survey of Multisystem Inflammatory Syndrome in Children Associated With Coronavirus Disease 2019 in Japan,” Journal of Clinical Immunology 45 (2024): 51. 39613902 10.1007/s10875-024-01845-z

[41] D. E. Roh , Y. T. Lim , J. E. Kwon , and Y. H. Kim , “Kawasaki Disease Following SARS‐CoV‐2 Infection: Stronger Inflammation With No Increase in Cardiac Complications,” Frontiers in Pediatrics 10 (2022): 1036306. 36467487 10.3389/fped.2022.1036306 PMC9714663

[42] C. de Cevins , M. Luka , N. Smith , et al., “A Monocyte/Dendritic Cell Molecular Signature of SARS‐CoV‐2‐Related Multisystem Inflammatory Syndrome in Children With Severe Myocarditis,” Med 2 (2021): 1072–1092.e7. 34414385 10.1016/j.medj.2021.08.002 PMC8363470

[43] M. Moreews , K. Le Gouge , S. Khaldi‐Plassart , et al., “Polyclonal Expansion of TCR Vβ21.3+ CD4+ and CD8+ T Cells Is a Hallmark of Multisystem Inflammatory Syndrome in Children,” Science Immunology 6 (2021): eabh1516. 34035116 10.1126/sciimmunol.abh1516 PMC8815705

[44] R. A. Porritt , L. Paschold , M. N. Rivas , et al., “HLA Class I‐Associated Expansion of TRBV11‐2 T Cells in Multisystem Inflammatory Syndrome in Children,” Journal of Clinical Investigation 131 (2021): e146614. 33705359 10.1172/JCI146614 PMC8121516

[45] A. Amphlett , “Far East Scarlet‐Like Fever: A Review of the Epidemiology, Symptomatology, and Role of Superantigenic Toxin: *Yersinia pseudotuberculosis*‐Derived Mitogen A,” Open Forum Infectious Diseases 3 (2016): ofv202. 26819960 10.1093/ofid/ofv202 PMC4728291

[46] K. Sato , K. Ouchi , and M. Taki , “ *Yersinia pseudotuberculosis* Infection in Children, Resembling Izumi Fever and Kawasaki Syndrome,” Pediatric Infectious Disease Journal 2 (1983): 123–126. 10.1097/00006454-198303000-00011 6344044

[47] M. Tahara , K. Baba , K. Waki , and Y. Arakaki , “Analysis of Kawasaki Disease Showing Elevated Antibody Titres of *Yersinia pseudotuberculosis* ,” Acta Paediatrica 95 (2006): 1661–1664. 17129979 10.1080/08035250600750080

[48] T. Horinouchi , K. Nozu , K. Hamahira , et al., “ *Yersinia pseudotuberculosis* Infection in Kawasaki Disease and Its Clinical Characteristics,” BMC Pediatrics 15 (2015): 177. 26561332 10.1186/s12887-015-0497-2 PMC4642785

[49] P. Vincent , E. Salo , M. Skurnik , H. Fukushima , and M. Simonet , “Similarities of Kawasaki Disease and *Yersinia pseudotuberculosis* Infection Epidemiology,” Pediatric Infectious Disease Journal 26 (2007): 629–631. 17596806 10.1097/INF.0b013e3180616d3c

[50] D. J. E. Marriott , S. Taylor , and D. C. Dorman , “ *Yersinia enterocolitica* Infection in Children,” Medical Journal of Australia 143 (1985): 489–492. 3877859 10.5694/j.1326-5377.1985.tb119908.x

[51] C. C. Feeney , O. A. Ajagbe , and M. Suryadevara , “ *Yersinia enterocolitica* Infection Presenting as Incomplete Kawasaki Disease: 2 Cases and a Review of the Literature,” Journal of the Pediatric Infectious Diseases Society 10 (2021): 217–219. 32083301 10.1093/jpids/piaa016

[52] K. Yasuoka , Y. Gotoh , I. Taniguchi , et al., “Genome Analysis of Japanese *Yersinia pseudotuberculosis* Strains Isolated From Kawasaki Disease Patients and Other Sources and Their Phylogenetic Positions in the Global *Y. pseudotuberculosis* Population,” Microbiology and Immunology 69 (2025): 182–190. 39780644 10.1111/1348-0421.13199 PMC11873759

[53] F. Collyn , H. Fukushima , C. Carnoy , M. Simonet , and P. Vincent , “Linkage of the Horizontally Acquired *ypm* and *pil* Genes in *Yersinia pseudotuberculosis* ,” Infection and Immunity 73 (2005): 2556–2558. 15784605 10.1128/IAI.73.4.2556-2558.2005 PMC1087444

[54] T. Bergsbaken , S. L. Fink , and B. T. Cookson , “Pyroptosis: Host Cell Death and Inflammation,” Nature Reviews Microbiology 7 (2009): 99–109. 19148178 10.1038/nrmicro2070 PMC2910423

[55] D. E. Min , D. H. Kim , M. Y. Han , S. H. Cha , and K. L. Yoon , “High Antistreptolysin O Titer Is Associated With Coronary Artery Lesions in Patients With Kawasaki Disease,” Korean Journal of Pediatrics 62 (2019): 235–239. 30404429 10.3345/kjp.2018.06989 PMC6584233

[56] Y. Tang , W. Yan , L. Sun , et al., “Kawasaki Disease Associated With *Mycoplasma pneumoniae* ,” Italian Journal of Pediatrics 42 (2016): 83. 27609267 10.1186/s13052-016-0292-1 PMC5016862

[57] M. Rani , S. E. Nicholson , Q. Zhang , and M. G. Schwacha , “Damage‐Associated Molecular Patterns (DAMPs) Released After Burn Are Associated With Inflammation and Monocyte Activation,” Burns 43 (2017): 297–303. 28341255 10.1016/j.burns.2016.10.001 PMC5373089

[58] H. Yanagawa , Y. Nakamura , M. Yashiro , et al., “A Nationwide Incidence Survey of Kawasaki Disease in 1985‐1986 in Japan,” Journal of Infectious Diseases 158 (1988): 1296–1301. 3198940 10.1093/infdis/158.6.1296

[59] L. Jiang , K. Tang , M. Levin , et al., “COVID‐19 and Multisystem Inflammatory Syndrome in Children and Adolescents,” Lancet Infectious Diseases 20 (2020): e276–e288. 32818434 10.1016/S1473-3099(20)30651-4 PMC7431129

[60] J. H. Lim , Y. K. Kim , S. H. Min , S. W. Kim , Y. H. Lee , and J. M. Lee , “Seasonal Trends of Viral Prevalence and Incidence of Kawasaki Disease: A Korea Public Health Data Analysis,” Journal of Clinical Medicine 10 (2021): 3301. 34362085 10.3390/jcm10153301 PMC8347058

[61] D. Aguirre , J. Cerda , C. Perret , A. Borzutzky , and R. Hoyos‐Bachiloglu , “Asociación temporal entre la circulación de virus respiratorios y hospitalizaciones por enfermedad de Kawasaki,” Revista Chilena de Infectología 38 (2021): 152–160. 34184704 10.4067/S0716-10182021000200152

[62] K. H. Rand , S. Bhaduri‐McIntosh , M. J. Gurka , X. Chi , and A. Harris , “Is Kawasaki Disease Caused by a Respiratory Virus?,” Pediatric Infectious Disease Journal 42 (2023): 468–472. 37171979 10.1097/INF.0000000000003889

[63] J. M. Kang , J. Jung , Y. E. Kim , et al., “Temporal Correlation Between Kawasaki Disease and Infectious Diseases in South Korea,” JAMA Network Open 5 (2022): e2147363. 35129593 10.1001/jamanetworkopen.2021.47363 PMC8822386

[64] O. Matveeva , Y. Nechipurenko , D. Lagutkin , Y. E. Yegorov , and J. Kzhyshkowska , “SARS‐CoV‐2 Infection of Phagocytic Immune Cells and COVID‐19 Pathology: Antibody‐Dependent as Well as Independent Cell Entry,” Frontiers in Immunology 13 (2022): 1050478. 36532011 10.3389/fimmu.2022.1050478 PMC9751203

[65] H. A. Rothan and S. N. Byrareddy , “The Potential Threat of Multisystem Inflammatory Syndrome in Children During the COVID‐19 Pandemic,” Pediatric Allergy and Immunology 32 (2021): 17–22. 32897642 10.1111/pai.13361 PMC7887110

[66] C. Junqueira , Â. Crespo , S. Ranjbar , et al., “FcγR‐Mediated SARS‐CoV‐2 Infection of Monocytes Activates Inflammation,” Nature 606 (2022): 576–584. 35385861 10.1038/s41586-022-04702-4 PMC10071495

[67] C. Zeng , R. Wang , and H. Tan , “Role of Pyroptosis in Cardiovascular Diseases and Its Therapeutic Implications,” International Journal of Biological Sciences 15 (2019): 1345–1357. 31337966 10.7150/ijbs.33568 PMC6643148

[68] J. A. Cerato , E. F. da Silva , and B. N. Porto , “Breaking Bad: Inflammasome Activation by Respiratory Viruses,” Biology (Basel) 12 (2023): 943. 37508374 10.3390/biology12070943 PMC10376673

[69] Q. Zheng , C. Hua , Q. Liang , and H. Cheng , “The NLRP3 Inflammasome in Viral Infection (Review),” Molecular Medicine Reports 28 (2023): 160. 37417336 10.3892/mmr.2023.13047 PMC10407610

[70] T. Hara , K. Furuno , K. Yamamura , et al., “Assessment of Pediatric Admissions for Kawasaki Disease or Infectious Disease During the COVID‐19 State of Emergency in Japan,” JAMA Network Open 4 (2021): e214475. 33822065 10.1001/jamanetworkopen.2021.4475 PMC8025113

[71] Z. Valtuille , A. Lefevre‐Utile , N. Ouldali , et al., “Calculating the Fraction of Kawasaki Disease Potentially Attributable to Seasonal Pathogens: A Time Series Analysis,” EClinicalMedicine 61 (2023): 102078. 37483549 10.1016/j.eclinm.2023.102078 PMC10359724

[72] L. L. DeHaan , C. D. Copeland , J. A. Burney , et al., “Age‐Dependent Variations in Kawasaki Disease Incidence in Japan,” JAMA Network Open 7 (2024): e2355001. 38319657 10.1001/jamanetworkopen.2023.55001 PMC10848069

[73] X. Rodó , J. Ballester , D. Cayan , et al., “Association of Kawasaki Disease With Tropospheric Wind Patterns,” Scientific Reports 1 (2011): 152. 22355668 10.1038/srep00152 PMC3240972

[74] D. Zhivaki and J. C. Kagan , “Innate Immune Detection of Lipid Oxidation as a Threat Assessment Strategy,” Nature Reviews Immunology 22 (2022): 322–330. 10.1038/s41577-021-00618-8 PMC8454293 34548649

[75] K. C. Barnett and J. C. Kagan , “Lipids That Directly Regulate Innate Immune Signal Transduction,” Innate Immunity 26 (2020): 4–14. 31180799 10.1177/1753425919852695 PMC6901815

[76] M. Ma , W. Jiang , and R. Zhou , “DAMPs and DAMP‐Sensing Receptors in Inflammation and Diseases,” Immunity 57 (2024): 752–771. 38599169 10.1016/j.immuni.2024.03.002

[77] S. Okada , H. Yasudo , Y. Ohnishi , et al., “Interleukin‐33/ST2 Axis as Potential Biomarker and Therapeutic Target in Kawasaki Disease,” Inflammation 46 (2023): 480–490. 36208354 10.1007/s10753-022-01753-7

[78] M. A. Beckley , S. Shrestha , K. K. Singh , and M. A. Portman , “The Role of Mitochondria in the Pathogenesis of Kawasaki Disease,” Frontiers in Immunology 13 (2022): 1017401. 36300112 10.3389/fimmu.2022.1017401 PMC9592088

[79] K. Wei , T. Chen , H. Fang , X. Shen , Z. Tang , and J. Zhao , “Mitochondrial DNA Release via the Mitochondrial Permeability Transition Pore Activates the cGAS‐STING Pathway, Exacerbating Inflammation in Acute Kawasaki Disease,” Cell Communication and Signaling 22 (2024): 328. 38872145 10.1186/s12964-024-01677-9 PMC11177463

[80] Y. Okuma , K. Suda , H. Nakaoka , et al., “Serum Tenascin‐C as a Novel Predictor for Risk of Coronary Artery Lesion and Resistance to Intravenous Immunoglobulin in Kawasaki Disease—A Multicenter Retrospective Study,” Circulation Journal 80 (2016): 2376–2381. 27746411 10.1253/circj.CJ-16-0563

[81] C. Jia , J. Zhang , H. Chen , et al., “Endothelial Cell Pyroptosis Plays An Important Role īn Kawasaki Disease via HMGB1/RAGE/Cathespin B Signaling Pathway and NLRP3 Inflammasome Activation,” Cell Death & Disease 10 (2019): 778. 31611559 10.1038/s41419-019-2021-3 PMC6791856

[82] S. J. Forrester , D. S. Kikuchi , M. S. Hernandes , Q. Xu , and K. K. Griendling , “Reactive Oxygen Species in Metabolic and Inflammatory Signaling,” Circulation Research 122 (2018): 877–902. 29700084 10.1161/CIRCRESAHA.117.311401 PMC5926825

[83] M. Tsuge , K. Uda , T. Eitoku , N. Matsumoto , T. Yorifuji , and H. Tsukahara , “Roles of Oxidative Injury and Nitric Oxide System Derangements in Kawasaki Disease Pathogenesis: A Systematic Review,” International Journal of Molecular Sciences 24 (2023): 15450. 37895129 10.3390/ijms242015450 PMC10607378

[84] V. Bochkov , B. Gesslbauer , C. Mauerhofer , M. Philippova , P. Erne , and O. V. Oskolkova , “Pleiotropic Effects of Oxidized Phospholipids,” Free Radical Biology and Medicine 111 (2017): 6–24. 28027924 10.1016/j.freeradbiomed.2016.12.034

[85] Y. E. He , H. X. Qiu , R. Z. Wu , et al., “Oxidised Low‐Density Lipoprotein and Its Receptor‐Mediated Endothelial Dysfunction Are Associated With Coronary Artery Lesions in Kawasaki Disease,” Journal of Cardiovascular Translational Research 13 (2020): 204–214. 31428922 10.1007/s12265-019-09908-y

[86] F. M. van der Valk , S. Bekkering , J. Kroon , et al., “Oxidized Phospholipids on Lipoprotein(a) Elicit Arterial Wall Inflammation and an Inflammatory Monocyte Response in Humans,” Circulation 134 (2016): 611–624. 27496857 10.1161/CIRCULATIONAHA.116.020838 PMC4995139

[87] Z. Zhaolin , C. Jiaojiao , W. Peng , et al., “OxLDL Induces Vascular Endothelial Cell Pyroptosis Through miR‐125a‐5p/TET2 Pathway,” Journal of Cellular Physiology 234 (2019): 7475–7491. 30370524 10.1002/jcp.27509

[88] T. Sawamura , N. Kume , T. Aoyama , et al., “An Endothelial Receptor for Oxidized Low‐Density Lipoprotein,” Nature 386 (1997): 73–77. 9052782 10.1038/386073a0

[89] D. A. Chistiakov , A. N. Orekhov , and Y. V. Bobryshev , “LOX‐1‐Mediated Effects on Vascular Cells in Atherosclerosis,” Cellular Physiology and Biochemistry 38 (2016): 1851–1859. 27160316 10.1159/000443123

[90] H. Kim , H. Jang , W. Lee , et al., “Association Between Long‐Term PM(2.5) Exposure and Risk of Kawasaki Disease in Children: A Nationwide Longitudinal Cohort Study,” Environmental Research 244 (2024): 117823. 38072109 10.1016/j.envres.2023.117823

[91] K. Yoneda , D. Shinjo , N. Takahashi , and K. Fushimi , “Spatiotemporal Analysis of the Association Between Kawasaki Disease Incidence and PM(2.5) Exposure: A Nationwide Database Study in Japan,” BMJ Paediatrics Open 8 (2024): e002887. 39327060 10.1136/bmjpo-2024-002887 PMC11428985

[92] Y. Inada , M. Sonoda , Y. Mizuno , et al., “CD14 Down‐Modulation as a Real‐Time Biomarker in Kawasaki Disease,” Clinical & Translational Immunology 13 (2024): e1482. 38162960 10.1002/cti2.1482 PMC10757666

[93] Y. Onouchi , T. Gunji , J. C. Burns , et al., “ITPKC Functional Polymorphism Associated With Kawasaki Disease Susceptibility and Formation of Coronary Artery Aneurysms,” Nature Genetics 40 (2008): 35–42. 18084290 10.1038/ng.2007.59 PMC2876982

[94] Y. Onouchi , K. Ozaki , J. C. Buns , et al., “Common Variants in CASP3 Confer Susceptibility to Kawasaki Disease,” Human Molecular Genetics 19 (2010): 2898–2906. 20423928 10.1093/hmg/ddq176 PMC2893807

[95] Y. Onouchi , K. Ozaki , J. C. Burns , et al., “A Genome‐Wide Association Study Identifies Three New Risk Loci for Kawasaki Disease,” Nature Genetics 44 (2012): 517–521. 22446962 10.1038/ng.2220

[96] Y. Onouchi , “The Genetics of Kawasaki Disease,” International Journal of Rheumatic Diseases 21 (2018): 26–30. 29152908 10.1111/1756-185X.13218

[97] H. C. Kuo , Y. W. Hsu , C. M. Wu , et al., “A Replication Study for Association of ITPKC and CASP3 Two‐Locus Analysis in IVIG Unresponsiveness and Coronary Artery Lesion in Kawasaki Disease,” PLoS One 8 (2013): e69685. 23894522 10.1371/journal.pone.0069685 PMC3722201

[98] C. C. Khor , S. Davila , W. B. Breunis , et al., “Genome‐Wide Association Study Identifies FCGR2A as a Susceptibility Locus for Kawasaki Disease,” Nature Genetics 43 (2011): 1241–1246. 22081228 10.1038/ng.981

[99] M. P. Alphonse , T. T. Duong , C. Shumitzu , et al., “Inositol‐Triphosphate 3‐Kinase C Mediates Inflammasome Activation and Treatment Response in Kawasaki Disease,” Journal of Immunology 197 (2016): 3481–3489. 10.4049/jimmunol.1600388 27694492

[100] S. S. Wright , C. Wang , A. Ta , et al., “A Bacterial Toxin Co‐Opts Caspase‐3 to Disable Active Gasdermin D and Limit Macrophage Pyroptosis,” Cell Reports 43 (2024): 114004. 38522070 10.1016/j.celrep.2024.114004 PMC11095105

[101] R. Chai , Y. Li , L. Shui , L. Ni , and A. Zhang , “The Role of Pyroptosis in Inflammatory Diseases,” Frontiers in Cell and Developmental Biology 11 (2023): 1173235. 37250902 10.3389/fcell.2023.1173235 PMC10213465

[102] J. C. Anania , A. M. Chenoweth , B. D. Wines , and P. M. Hogarth , “The Human FcγRII (CD32) Family of Leukocyte FcR in Health and Disease,” Frontiers in Immunology 10 (2019): 464. 30941127 10.3389/fimmu.2019.00464 PMC6433993

[103] S. Bournazos , A. Gupta , and J. V. Ravetch , “The Role of IgG Fc Receptors in Antibody‐Dependent Enhancement,” Nature Reviews Immunology 20 (2020): 633–643. 10.1038/s41577-020-00410-0 PMC7418887 32782358

[104] S. Frampton , R. Smith , L. Ferson , et al., “Fc Gamma Receptors: Their Evolution, Genomic Architecture, Genetic Variation, and Impact on Human Disease,” Immunological Reviews 328 (2024): 65–97. 39345014 10.1111/imr.13401 PMC11659932

[105] F. Harada , M. Sada , T. Kamiya , Y. Yanase , T. Kawasaki , and T. Sasazuki , “Genetic Analysis of Kawasaki Syndrome,” American Journal of Human Genetics 39 (1986): 537–539. 3766546 PMC1683975

[106] A. Kottek , C. Shimizu , and J. C. Burns , “Kawasaki Disease in Monozygotic Twins,” Pediatric Infectious Disease Journal 30 (2011): 1114–1116. 21796015 10.1097/INF.0b013e31822ac4ff PMC3222730

[107] Z. Wang , L. Xie , G. Ding , et al., “Single‐Cell RNA Sequencing of Peripheral Blood Mononuclear Cells From Acute Kawasaki Disease Patients,” Nature Communications 12 (2021): 5444. 10.1038/s41467-021-25771-5 PMC8440575 34521850

[108] L. Chen , X. Long , Q. Xu , et al., “Elevated Serum Levels of S100A8/A9 and HMGB1 at Hospital Admission Are Correlated With Inferior Clinical Outcomes in COVID‐19 Patients,” Cellular & Molecular Immunology 17 (2020): 992–994. 32620787 10.1038/s41423-020-0492-x PMC7332851

[109] D. Tang , P. Comish , and R. Kang , “The Hallmarks of COVID‐19 Disease,” PLoS Pathogens 16 (2020): e1008536. 32442210 10.1371/journal.ppat.1008536 PMC7244094

[110] S. Lee , R. Channappanavar , and T. D. Kanneganti , “Coronaviruses: Innate Immunity, Inflammasome Activation, Inflammatory Cell Death, and Cytokines,” Trends in Immunology 41 (2020): 1083–1099. 33153908 10.1016/j.it.2020.10.005 PMC7561287

[111] K. C. Kuo , Y. L. Yang , M. H. Lo , et al., “Increased Expression of Pyroptosis in Leukocytes of Patients With Kawasaki Disease,” Diagnostics 11 (2021): 2035. 34829381 10.3390/diagnostics11112035 PMC8620614

[112] Y. Xie and B. Han , “Exploring the Relationship Between Pyroptosis, Infiltrating Immune Cells and Kawasaki Disease With Resistance to Intravenous Immunoglobulin (IVIG) via Bioinformatic Analysis,” Immunobiology 227 (2022): 152261. 36029669 10.1016/j.imbio.2022.152261

[113] Z. Wen , Y. Xia , Y. Zhang , et al., “SIGIRR‐Caspase‐8 Signaling Mediates Endothelial Apoptosis in Kawasaki Disease,” Italian Journal of Pediatrics 49 (2023): 2. 36600293 10.1186/s13052-022-01401-8 PMC9811794

[114] M. Li , D. Liu , Z. Cheng , et al., “Serum NLRP3: A Potential Marker for Identifying High‐Risk Coronary Arterial Aneurysm in Children With Kawasaki Disease,” Cytokine 180 (2024): 156667. 38857561 10.1016/j.cyto.2024.156667

[115] S. Hirata , Y. Nakamura , and H. Yanagawa , “Incidence Rate of Recurrent Kawasaki Disease and Related Risk Factors: From the Results of Nationwide Surveys of Kawasaki Disease in Japan,” Acta Paediatrica 90 (2001): 40–44. 11227331 10.1080/080352501750064851

[116] R. A. Maddox , R. C. Holman , R. Uehara , et al., “Recurrent Kawasaki Disease: USA and Japan,” Pediatrics International 57 (2015): 1116–1120. 26096590 10.1111/ped.12733 PMC4676732

[117] M. G. Netea , J. Domínguez‐Andrés , L. B. Barreiro , et al., “Defining Trained Immunity and Its Role in Health and Disease,” Nature Reviews Immunology 20 (2020): 375–388. 10.1038/s41577-020-0285-6 PMC7186935 32132681

[118] Y. Shao , J. Saredy , W. Y. Yang , et al., “Vascular Endothelial Cells and Innate Immunity,” Arteriosclerosis, Thrombosis, and Vascular Biology 40 (2020): e138–e152. 32459541 10.1161/ATVBAHA.120.314330 PMC7263359

[119] M. Di Gioia , R. Spreafico , J. R. Springstead , et al., “Endogenous Oxidized Phospholipids Reprogram Cellular Metabolism and Boost Hyperinflammation,” Nature Immunology 21 (2020): 42–53. 31768073 10.1038/s41590-019-0539-2 PMC6923570

[120] K. E. Brodeur , M. Liu , D. Ibanez , et al., “Elevation of IL‐17 Cytokines Distinguishes Kawasaki Disease From Other Pediatric Inflammatory Disorders,” Arthritis & Rheumatology 76 (2024): 285–292. 37610270 10.1002/art.42680 PMC10842426

[121] V. Navarro‐Compán , L. Puig , S. Vidal , et al., “The Paradigm of IL‐23‐Independent Production of IL‐17F and IL‐17A and Their Role in Chronic Inflammatory Diseases,” Frontiers in Immunology 14 (2023): 1191782. 37600764 10.3389/fimmu.2023.1191782 PMC10437113

[122] M. E. Lindquist and M. D. Hicar , “B Cells and Antibodies in Kawasaki Disease,” International Journal of Molecular Sciences 20 (2019): 1834. 31013925 10.3390/ijms20081834 PMC6514959

[123] F. D. Faro and J. C. Burns , “Lipid Peroxidation and Kawasaki Disease,” Pediatric Cardiology 24 (2003): 611–612. 12784125 10.1007/s00246-002-0457-0

[124] Z. Kano , Y. Mizuno , K. Murata , et al., “Anti‐Oxidized Low‐Density Lipoprotein Antibodies Before and After Intravenous Immunoglobulin Therapy in Kawasaki Disease—Evidence for a Potentially Protective Role,” Circulation Reports 7 (2025): 359–364. 40352122 10.1253/circrep.CR-25-0018 PMC12061508

[125] R. Wu , Y. Shoenfeld , Y. Sherer , et al., “Anti‐Idiotypes to Oxidized LDL Antibodies in Intravenous Immunoglobulin Preparations—Possible Immunomodulation of Atherosclerosis,” Autoimmunity 36 (2003): 91–97. 12820691 10.1080/0891693031000080228

[126] D. Sudo , N. Makino , and Y. Nakamura , “Recurrent Kawasaki Disease and Cardiac Complications: Nationwide Surveys in Japan,” Archives of Disease in Childhood 105 (2020): 848–852. 32107252 10.1136/archdischild-2019-317238

[127] K. Murata , S. Onoyama , K. Yamamura , et al., “Kawasaki Disease and Vaccination: Prospective Case‐Control and Case‐Crossover Studies Among Infants in Japan,” Vaccines 9 (2021): 839. 34451964 10.3390/vaccines9080839 PMC8402330

[128] J. Stowe , N. J. Andrews , P. J. Turner , and E. Miller , “The Risk of Kawasaki Disease After Pneumococcal Conjugate & Meningococcal B Vaccine in England: A Self‐Controlled Case‐Series Analysis,” Vaccine 38 (2020): 4935–4939. 32536544 10.1016/j.vaccine.2020.05.089

[129] C. J. Binder , S. Hörkkö , A. Dewan , et al., “Pneumococcal Vaccination Decreases Atherosclerotic Lesion Formation: Molecular Mimicry Between *Streptococcus pneumoniae* and Oxidized LDL,” Nature Medicine 9 (2003): 736–743. 10.1038/nm876 12740573

[130] S. Ren , P. M. Hansbro , W. Srikusalanukul , et al., “Generation of Cardio‐Protective Antibodies After Pneumococcal Polysaccharide Vaccine: Early Results From a Randomised Controlled Trial,” Atherosclerosis 346 (2022): 68–74. 35290813 10.1016/j.atherosclerosis.2022.02.011

[131] P. N. Jone , A. Tremoulet , N. Choueiter , et al., “Update on Diagnosis and Management of Kawasaki Disease: A Scientific Statement From the American Heart Association,” Circulation 150 (2024): e481–e500. 39534969 10.1161/CIR.0000000000001295

[132] C. Galeotti , S. V. Kaveri , and J. Bayry , “IVIG‐Mediated Effector Functions in Autoimmune and Inflammatory Diseases,” International Immunology 29 (2017): 491–498. 28666326 10.1093/intimm/dxx039

[133] E. E. Perez , J. S. Orange , F. Bonilla , et al., “Update on the Use of Immunoglobulin in Human Disease: A Review of Evidence,” Journal of Allergy and Clinical Immunology 139 (2017): S1–S46. 28041678 10.1016/j.jaci.2016.09.023

[134] E. V. Sidorin and T. F. Solov'eva , “IgG‐Binding Proteins of Bacteria,” Biochemistry (Moscow) 76 (2011): 295–308. 21568864 10.1134/s0006297911030023

[135] T. Kusuda , Y. Nakashima , K. Murata , et al., “Kawasaki Disease‐Specific Molecules in the Sera Are Linked to Microbe‐Associated Molecular Patterns in the Biofilms,” PLoS One 9 (2014): e113054. 25411968 10.1371/journal.pone.0113054 PMC4239021

[136] D. Okuzaki , K. Ota , S. Takatsuki , et al., “FCN1 (M‐Ficolin), Which Directly Associates With Immunoglobulin G1, Is a Molecular Target of Intravenous Immunoglobulin Therapy for Kawasaki Disease,” Scientific Reports 7 (2017): 11334. 28900133 10.1038/s41598-017-11108-0 PMC5595863

[137] A. E. Coutinho and K. E. Chapman , “The Anti‐Inflammatory and Immunosuppressive Effects of Glucocorticoids, Recent Developments and Mechanistic Insights,” Molecular and Cellular Endocrinology 335 (2011): 2–13. 20398732 10.1016/j.mce.2010.04.005 PMC3047790

[138] K. Ueno , Y. Nomura , Y. Morita , and Y. Kawano , “Prednisolone Suppresses the Extracellular Release of HMGB‐1 and Associated Inflammatory Pathways in Kawasaki Disease,” Frontiers in Immunology 12 (2021): 640315. 34079539 10.3389/fimmu.2021.640315 PMC8165186

[139] T. Horiuchi , H. Mitoma , S. Harashima , H. Tsukamoto , and T. Shimoda , “Transmembrane TNF‐α: Structure, Function and Interaction With Anti‐TNF Agents,” Rheumatology 49 (2010): 1215–1228. 20194223 10.1093/rheumatology/keq031 PMC2886310

[140] R. Mouy , J. L. Stephan , P. Pillet , E. Haddad , P. Hubert , and A. M. Prieur , “Efficacy of Cyclosporine A in the Treatment of Macrophage Activation Syndrome in Juvenile Arthritis: Report of Five Cases,” Journal of Pediatrics 129 (1996): 750–754. 8917244 10.1016/s0022-3476(96)70160-9

[141] K. Bendickova , F. Tidu , and J. Fric , “Calcineurin‐NFAT Signalling in Myeloid Leucocytes: New Prospects and Pitfalls in Immunosuppressive Therapy,” EMBO Molecular Medicine 9 (2017): 990–999. 28606994 10.15252/emmm.201707698 PMC5538425

[142] T. P. P. van den Bosch , N. M. Kannegieter , D. A. Hesselink , C. C. Baan , and A. T. Rowshani , “Targeting the Monocyte‐Macrophage Lineage in Solid Organ Transplantation,” Frontiers in Immunology 8 (2017): 153. 28261211 10.3389/fimmu.2017.00153 PMC5312419

[143] M. Mori , T. Imagawa , S. Katakura , et al., “Efficacy of Plasma Exchange Therapy for Kawasaki Disease Intractable to Intravenous Gamma‐Globulin,” Modern Rheumatology 14 (2004): 43–47. 17028804 10.1007/s10165-003-0264-3

[144] T. Fujimaru , S. Ito , H. Masuda , et al., “Decreased Levels of Inflammatory Cytokines in Immunoglobulin‐Resistant Kawasaki Disease After Plasma Exchange,” Cytokine 70 (2014): 156–160. 25082649 10.1016/j.cyto.2014.07.003

[145] J. Abe , B. L. Kotzin , K. Jujo , et al., “Selective Expansion of T Cells Expressing T‐Cell Receptor Variable Regions V Beta 2 and V Beta 8 in Kawasaki Disease,” Proceedings of the National Academy of Sciences of the United States of America 89 (1992): 4066–4070. 1315049 10.1073/pnas.89.9.4066 PMC525633

[146] A. H. Rowley , S. C. Baker , S. T. Shulman , et al., “Detection of Antigen in Bronchial Epithelium and Macrophages in Acute Kawasaki Disease by Use of Synthetic Antibody,” Journal of infectious diseases 190 (2004): 856–865. 15272416 10.1086/422648

[147] A. H. Rowley , S. C. Baker , S. T. Shulman , et al., “Ultrastructural, Immunofluorescence, and RNA Evidence Support the Hypothesis of a “New” Virus Associated With Kawasaki Disease,” Journal of Infectious Diseases 203 (2011): 1021–1030. 21402552 10.1093/infdis/jiq136 PMC3068030

[148] A. H. Rowley , R. Byrd , D. Arrollo , et al., “Monoclonal Antibodies From Children With Acute Kawasaki Disease Identify a Common Antigenic Target in Fatal Cases Over 5 Decades,” Laboratory Investigation 105 (2025): 104131. 40122524 10.1016/j.labinv.2025.104131 PMC12822914

[149] P. Yang , J. Zhang , K. Zhang , et al., “Prenatal and Postnatal Ambient Air Pollution and Kawasaki Disease,” JACC: Advances 4 (2025): 101651. 40088736 10.1016/j.jacadv.2025.101651 PMC11937670

